# “It All Exists Within Me”: A Qualitative Study Exploring Mental Health Service Experiences in Adults With Comorbid Eating Disorders and Emotionally Unstable Personality Disorder

**DOI:** 10.1002/jclp.70221

**Published:** 2026-09-12

**Authors:** Amy Lunn, Angie Jenkin, Alex Lau‐Zhu, Lisle Scott, Megan Dunnage, John Stewart, Joanna Holliday

**Affiliations:** ^1^ Oxford Health NHS Foundation Trust & Oxford Institute of Clinical Psychology Training and Research Oxford UK; ^2^ Oxford Institute of Clinical Psychology Training and Research Oxford UK; ^3^ Oxford Department of Experimental Psychology Oxford UK; ^4^ Department of Brain Sciences Imperial College London UK; ^5^ Oxford Health Foundation trust Linacre College UK; ^6^ Oxford Health NHS Foundation Trust Oxford UK; ^7^ South London and Maudsley NHS Foundation Trust UK

## Abstract

**Objective:**

Comorbidity between Eating Disorders (ED) and Emotionally Unstable Personality Disorder (EUPD) is high. This comorbidity is also linked with worse outcomes for traditional evidence‐based treatments. We aimed to improve mental health service experience for adults with comorbid EUPD and ED by: (1) understanding the service‐user experience of receiving and accessing care; (2) identifying areas of unmet need; and (3) creating recommendations for improving care for this population.

**Methods:**

The project comprised semi‐structured interviews with nine service‐users within the UK National Health Service. Reflexive Thematic Analysis was used to generate themes, which informed clinical recommendations and a framework for improving care to meet local needs, with implications beyond.

**Results:**

Service‐users described long waits and disjointed care, often feeling pressured to prioritise one difficulty over another in ways that did not align with their own goals. Many reported that their needs were only recognised at crisis point, highlighting a gap between clinician understanding and personal experiences. Six themes were derived from patient interviews. Psychoeducation about the links between EUPD and ED was seen as helpful, as well as the need for more consistent individualised care that better reflects service‐users’ goals and needs.

**Conclusions:**

The project increased understanding of the experiences of mental health service use in adults with comorbid ED and EUPD, and produced a set of recommendations, aimed at improving care for these individuals. These included a thorough assessment of comorbidity, consideration of service‐user choice, alongside appropriate service adaptations to best meet service‐users’ goals and needs.

## 1 Introduction

Emotionally Unstable Personality Disorder (EUPD) increases the likelihood of experiencing an eating disorder (ED), poor treatment outcomes for ED recovery, and suicide risks. Increased rates of Anorexia Nervosa (AN) have also been observed in EUPD populations, with AN prevalence rates estimated to be 21%–40% (Kountza et al. 2018; Martinussen et al. 2017), and 16%–25% for AN, binge eating/purging subtype (Farstad et al. 2016; Sansone and Sansone 2011).

The presence of EUPD can complicate treatment for ED; specific EUPD‐traits are thought to reduce treatment efficacy and engagement, by impeding capacity to adhere to therapeutic goals, structure and homework (Constantino et al. 2011; Davidson 2000; Martinussen et al. 2017). An increased level of rigidity and inflexibility is further linked to EUPD‐related difficulties (Smith et al. 2017), and those with EUPD are more likely to report negative treatment experiences (Johansen et al. 2013). The increased level of complexity associated with comorbid EUPD and ED, highlights the imperative need to better understand the comorbidity between EUPD and ED to improve outcomes for this population.

Dialectical Behaviour Therapy (DBT) is an evidence‐based treatment for EUPD, which has demonstrated effectiveness in treating emotional dysregulation and suicidality in those with EUPD (Hernandez‐Bustamante et al. 2024). DBT seeks to address the biopsychosocial drivers underpinning EUPD (Linehan 1993). A recent review on DBT for ED indicated that it can also be effective in improving emotion regulation and overall ED psychopathology (Rozakou‐Soumalia et al. 2021); however, individuals with comorbid EUPD and ED were not included.

Of the literature including participants with comorbid EUPD and an ED, mixed treatment outcomes are apparent. Denning et al. (2023) employed DBT, finding initial improvements in ED and suicidal behaviours. Nevertheless, results indicated a low proportion of ED remission amongst the sample, following intervention (Denning et al. 2023). Federici (2010) found improvements for suicidal behaviour following DBT, although no significant differences on key ED domains, such as binge‐eating or vomiting. There is also limited research investigating use of CBT with EUPD and ED populations. Some studies show initial improvement of ED and EUPD symptoms (Park et al. 2014), but lack follow‐up data. Two further studies have also demonstrated change in ED and personality characteristics (Rowe et al. 2008; Thompson‐Brenner et al. 2016), although low remission is noted at 55.2% for binge‐eating and purging behaviours (Thompson‐Brenner et al. 2016). Overall, literature employing traditional evidence‐based models is limited, and lacks consistent outcomes.

A small body of literature has demonstrated good outcomes for individuals with comorbid EUPD and EDs for adapted evidence‐based models, such as DBT, with an incorporated emphasis on ED symptomatology (Ben‐Porath et al. 2009; Chen et al. 2008; Kröger et al. 2010; Palmer et al. 2003; Salbach‐Andrae et al. 2008), including use of Multi‐Diagnostic Eating Disorder DBT (MED‐DBT; Federici and Wisniewski 2013). All papers observed significant reductions in ED symptoms, and suicidal behaviours/self‐injury, including in studies that provided follow‐up data (Chen et al. 2008; Kröger et al. 2010; Palmer et al. 2003).

Despite adapted evidence‐based models demonstrating preferential outcomes, treatment outcomes for this population are largely mixed. Moreover, wider literature lacks an understanding of service‐user experiences of accessing care and service‐user‐valued outcomes. In the UK, national guidelines recommend joint‐working between EUPD and ED teams for individuals presenting with comorbid needs (National Colloborating Centre for Mental Health 2019), however, there remains little guidance on how clinicians can do so in a holistic, needs‐driven manner. Services also often comprise diagnostic‐led pathways, which have been suggested to pose the risk of care becoming fragmented and not effectively meeting population needs (National Colloborating Centre for Mental Health 2019). Understanding service‐user views of accessing and receiving care, is imperative in improving care within the NHS.

### 1.1 Theoretical Basis

Linehan's Biosocial Model (Linehan 1993) provides a useful framework to understand the development of comorbid EUPD and ED. According to this model, individuals with EUPD have a combination of biological (e.g. genetic differences in impulsivity and reactivity) and social vulnerabilities (e.g. an invalidating developmental context), which lead to: (1) increased sensitivity to emotion; (2) an inability to regulate intense emotional responses; and, (3), a delayed return to emotional baseline (Crowell et al. 2009). The invalidating environment includes a caregiver's intolerance towards emotional expression, abuse, or neglect, which can prevent the child from learning to label, regulate, tolerate, and/or understand emotional responses. This can result in oscillation between emotional inhibition and extreme emotional lability, including dysfunctional responses to attempt to cope, such as self‐harm, increased impulsivity and dysregulated eating.

The interaction of biological vulnerabilities (emotional, nutritional, or both) and an invalidating environment are also implicated in the development of disordered eating (Wisniewski and Kelly 2003). Difficulties in regulating emotions, avoidance and control of emotion, have been found in individuals with AN (Casper et al. 1992), Bulimia Nervosa (BN) and Binge Eating Disorder (BED; Telch and Agras 1996). Invalidating environments are also often reported in individuals with ED (Ford et al. 2011; Waller et al. 2007). Taken together, the presence of comorbid ED and EUPD may indicate general deficits in emotion dysregulation, with disordered eating used to cope with, or regulate intense emotions.

While this theory provides a useful framework for conceptualising the development of a comorbid EUPD and ED, the needs and views of this population from the perspective of experts by experience remain little explored.

### 1.2 Aim and Objectives of the Project

Our overarching aim was to understand how mental health service experiences can be improved at a local service for individuals with comorbid EUPD and ED. Specific objectives are:

1. To understand the service‐user experience of seeking, accessing and receiving care.
2. To understand areas of unmet need.
3. To create recommendations for improving care.

## 2 Methods

### 2.1 The Service Context

Within the local NHS context, adult ED services consist of community ED teams (A‐ED) with access to inpatient treatment when required. Complex emotional needs (CEN; a term often used interchangeable with EUPD) service is an adult outpatient service offering support for individuals with EUPD. For individuals presenting with comorbid ED and CEN/EUPD, joint working between both the A‐ED and CEN is advocated for (National Colloborating Centre for Mental Health 2019). However, staff report constraints in accessing evidence‐based interventions. For example, a low BMI can pose a barrier to engaging with DBT; conversely, therapy‐interfering behaviours (TIB), such as self‐injury, present barriers to engaging with ED interventions, such as CBT‐E. Consequently, care of this dual‐diagnosis population can remain fragmented and inconsistent.

### 2.2 Participants

Service‐users were eligible if they were identified as having diagnoses of both EUPD and an ED or experiences of accessing services for needs related to both even in the absence of formal diagnoses. This is because of recognised challenges of misdiagnosis and under diagnosis between these conditions.

Eligible participants (*n* = 9) were recruited using purposive sampling. Figure 1 details the participant recruitment process. Sample size was determined by the specificity of the sample, the focus of the research questions and information power; nine participants provided sufficient depth and richness to meaningfully address the research questions. We prioritised the generation of an information‐rich dataset that aligned with the conceptual and methodological coherence of the data analytical approach employed. Practical considerations such as participant availability also informed the final sample. Demographic information and waiting times were gathered from routine service data and can be found in Table 1.

**Figure 1:**
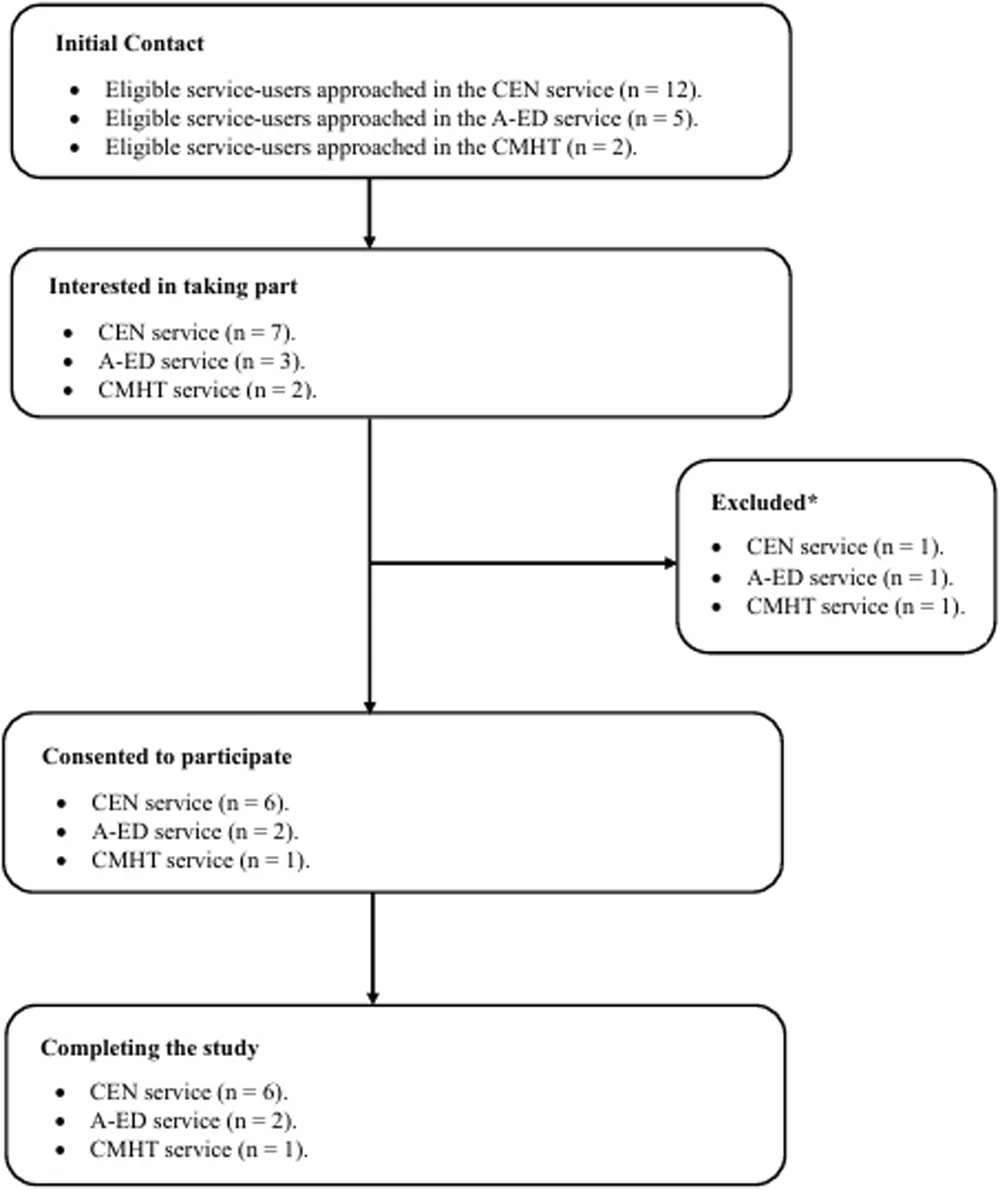
Participant recruitment flowchart. Acronyms used: A‐ED, Adults Eating Disorders; CEN, Complex Emotional Needs; CMHT, Community Mental Health Team. *Participants who declined to continue participation due to time constraints or personal circumstances.

**Table 1:** Participant demographics.

Demographic variable	Mean (M) and SD or *n* and %	Range
Age (years)	*M* = 29.2 (11.3)	22–55
Ethnicity	White British (*n* = 9; 100%)	
Gender	Female (*n* = 9; 100%)	
Length of ED since onset (years)	*M* = 15 (10.1)	7–40 years
Eating disorder diagnoses	Anorexia Nervosa (*n* = 3; 30%)	
	Atypical Anorexia Nervosa (*n* = 2; 22.2%)	
	Anorexia Nervosa, later developed to Binge‐eating Disorder (*n* = 1; 11.1%)	
	Bulimia Nervosa (*n* = 1; 11.1%)	
	Otherwise Specified Feeding and Eating Disorder (*n* = 1; 11.1%)	
	Disordered eating (*n* = 1; 11.1%)	
Length of EUPD since onset (years)	*M* = 15.6 years (11.1)	6–40 years
Other diagnoses	Complex posttraumatic stress disorder (*n* = 3; 30%)	
	Depression and anxiety (*n* = 2; 22.2%)	
	Posttraumatic stress disorder (*n* = 1; 11.1%)	
	Autism (*n* = 1; 11.1%)	
	No other diagnoses (*n* = 2; 22.2%).	
Current service	CEN (*n* = 6; 66.6%)	
	A‐ED (*n* = 2; 22.2%)	
	CMHT (*n* = 1; 11.1%)	
Total waiting time for A‐ED service (years)	*M* = 2 years^a^	2 years^a^
Total waiting time for CEN service (years)	*M* = 2.8 years (0.84)	2–4 years^b^
Total waiting time across community mental health services (years)	*M* = 4.8 years (2.22)	3–10 years

^a^ Data derived from *n* = 4/9 participants. Other participants had not yet been referred (*n* = 1) or received hospitalisation as their first point of ED care (*n* = 4).

^b^ Data derived from 8/9 participants. At the time of data collection, one participant continued to be waiting for 4 years.

Abbreviations: A‐ED, Adults Eating Disorders; CEN, Complex Emotional Needs; CMHT, Community Mental Health Team; ED, Eating Disorder; EUPD, Emotionally Unstable Personality Disorder.

### 2.3 Design and Procedure

A qualitative research design was employed. We conducted semi‐structured interviews with service‐users between May to July 2024. Eligible participants were approached by their assigned clinician to ascertain interest in taking part in the study. Patients expressing interest were provided with the information sheet and consent form and were contacted by the lead researcher to arrange an interview (Figure 1). All service‐users provided informed consent online prior to the interviews.

### 2.4 Interviews

A semi‐structured interview schedule, comprising open‐ended questions and prompts, was used as the primary data collection tool. Development of the schedule had input from a lived‐experience consultant. Where participants expressed a preference, written versions of the interview questions were provided in addition to the oral format. Interviews were conducted over videocall, audio recorded and transcribed.

Interviews were conducted by the lead author, who completed a bracketing interview prior to the first interview and kept a reflexive record of observations and biases throughout.

### 2.5 Ethics

The project was approved by the local Research and Development Team. All service‐users who met provisional criteria for the study were approached by their care team about participating.

### 2.6 Data Analysis

Data were managed and coded using NVivo 14 (Lumivero 2023). Data were analysed using Reflexive Thematic Analysis (TA; Braun and Clarke 2006). Our epistemological and ontological approach was a form of constructivism, as we looked for participants truth or reality, while acknowledging the project was influenced by the UK NHS structure, in which it was situated. Participants with divergent experiences were included.

Semantic (descriptive) and latent (implicit) coding was used, and the codes were collated to develop themes and subthemes in response to the research question. The lead researcher transcribed and analysed all data. The lead researcher had no prior relationship with participants and maintained a reflexive stance throughout data collection and analysis. While not central to Reflexive TA, the second and fourth author also coded the first two transcripts independently, to support synthesis of themes. Where possible, the words used by service‐users were directly included within theme titles.

## 3 Results

Six themes were identified in the service‐user interviews. Theme descriptions and subthemes are outlined in Table 2.

**Table 2:** Thematic analysis results from interview data.

Theme and Description	Subthemes	Illustrative Quotes
**Theme 1: Navigating diagnosis: The quest for understanding and effective support** This theme captures the perceived importance of receiving “answers,” and the perception that diagnosis will provide exploration and access to care	1.1 Perception of diagnosis as a means to exploration and access to the “right care.” 1.2 The need for education and exploration post‐diagnosis 1.3. The intersection between ED and EUPD: “how it fits together.”	Participant001: “They weren't really interested in diagnosing which type of personality disorder it was… which was a problem for me, because in order to understand myself, I wanted to know what it was”. Participant001: “I ended up going private, and that was when I finally got the diagnosis, and then people started taking me seriously because I had the diagnosis.” Participant006: “After I was assessed, I didn't get much information on the diagnosis [from the ED team]. I wanted more information on where to start, to help myself a little. Because it felt like I got a diagnosis and then was just of left to it, as I was under complex needs.” Participant004: “Like finally having someone to talk to, and professionals actually listening. Just actually getting an answer and some help was massive.”
**Theme 2: Experiences of siloed care: “It's either the EUPD or the ED”: experiences of disconnect and misplaced focus.** This theme reflections the notion that service‐users often felt compelled to choose what they wanted help with, which conflicted with their own views and resulted in a sense of disjointed care.	2.1. Lack of holistic care. 2.2. Unexplored comorbidity. 2.3. An overemphasis on risk and symptoms. 2.4. Conflicting professional understanding.	Participant007: “There's a lot of focus on like coping skills and crisis plans, which at the time is good when you are in a crisis… but if we just focus on managing the symptoms, you don't really understand what caused it in the first place, so you just keep going back to those symptoms”. Participant008: I just I think there wasn't much communication between different services or just different people? It was very much focused on risk, which I get that they have to do that. But it was very much like what's the biggest risk at this point, and then I'm just gonna send you to these different people to help deal with that risk, but then nothing ever really, I don't know. It felt like it wasn't very, like a clear plan. It was very much just, oh, we'll just kind of send her around and hope that someone can help. Participant007: “they've been treated very separately, and I think that's kind of what was missing. I think that kind of could reduce the amount of time you spend in treatment or get in and out of hospitals.” Participant004: I know I can e‐mail, someone in my service about it, but I find it really hard to do that because, my eating difficulties haven't been spoken about, so I don't necessarily know how to speak about it. Participant001: I also had this eating disorder going on, but it was felt, I think, by the teams at the adult mental health that that was probably related to the personality disorder. But I felt it was more there in its own right as a separate thing.
**Theme 3: Crisis as a catalyst for support: “You have to be bad enough to get care.”** This theme captures the idea that experiencing a crisis often leads to receiving care, and also reflects the notion that worsening conditions or waiting can lead to further decline before help is provided.	3.1. Crisis as a gateway. 3.2. Motivated to meet the “crisis” threshold to receive care. 3.3. Delayed identification of difficulties.	Participant004: “My first emotion, when I was in A&E, I remember being very, very scared, and then really angry, it took way too long for me to find out what was going on, and took me ending up in hospital.” Participant001: You shouldn't have to hit crisis point in my eyes before you get the proper input because it's almost gone too far. By that point, you should, you know, be able to capture the person, as I say, when they're seeking the services, because that asking for help, so they know there's a problem then. Participant009: They've got to prioritise people who are struggling more, but then yeah, you don't, you don't feel like. Yeah, it kind of then, well, badly, it motivates you to want to get worse so that you'll get help, if that makes sense.
**Theme 4: Lost in the system: Waiting times and professional uncertainty** This theme highlights the difficulty in navigating the NHS structure to find the “right care,” and complexities around accessing treatment from differing pathways.	4.1. Navigating the system 4.2. Waiting times 4.3. Self‐advocacy in the face of being bounced around. 4.4 Experiences of professional labelling	Participant002: So when I was first put on the waiting list for complex needs, I was well enough to have benefitted from the service. Now, if they contact eating disorders and see what my BMI is, they're not even going to accept me. So, I will have waited four years for nothing. My peer worker said to me that they only need to take one look at me and they wouldn't accept me anyway. Participant001: I didn't know if I was sort of slipping through the net. Participant009: “I think sometimes professionals forget that you as like an outsider, you don't know how the system works, when I went to see the adult mental health team, they said, we can't help you, but complex needs can. And that felt like a huge blow, because I was like, I don't understand what any of this means. It's hard to know like the pathways.” Participant003: 003: All the fighting or the bouncing around between services, it's seems to stand to reason that that it's gonna exacerbate issues, and I think have therefore become more complicated than I was a few years back, so, if I just left things, then, you know, it would be like, possibly even more years of of that and or possibly I wouldn't be here.
**Theme 5: What makes care meaningful.** This theme encapsulates aspects of clinical practice that have been meaningful from a service‐user perspective.	5.1. Collaborative and supportive teams 5.2. Empathetic professionals 5.3. Having a consistent clinician	Participant001: I think any individual I've come across has been very nice, you know, like nice to deal with pleasant, understanding, empathetic. So there's definitely the right sorts of people in the roles. Participant005: Complex needs, it's probably the best experience I've had within the within any service that I've encountered. … I do feel like the people that work within the complex needs service or the people that I have met have just been very professional, but real people, but have kept it at a level where, you just ‐ they've created this sort of safe space for all of us to feel like, you know, we're all part of something and, we're all wanted within the group and valued within the group, which is really lovely to feel that way. Participant007: Having a care coordinator has also been helpful ‘cause, she's like the person that could refer you to different places if you need it or change on, like help change your medication or something. So that's also been helpful. I don't really know what else I'd do otherwise.
**Theme 6: Need for an integrated approach: “It all exists within me.”** This theme reflects the perceived interplay between EUPD and ED from a service‐user perspective, and insights into how care could better‐understand service‐user experiences.	6.1. The service‐user view of their comorbidity. 6.2. Understanding how they ‘go together’. 6.3. Going beyond physical risk.	Participant005: When things go a bit pear shaped with my mental health, I do find that it's [eating disorder] still a bit of a crutch for me to to fall back on that path. So even though I would say that I'm relatively recovered, there's still something there. ^±^ Participant009: I think like I have kind of like a very disordered view of eating, it's very and I think this is part of my personality difficulties, as I see it, very like black and white like. One one moment I'll be like, I can't eat anything because everything will make me fat or yeah and so it'll be like I'll go from not eating anything all day to then eating loads of things in the evening and I don't feel like I've had. Yeah, any support with it yet? Any kind of eating stuff. Participant002: I thought I could starve the evil bit of me out. I thought if I was smaller, there would be less for people to dislike. I never wanted to be slim and beautiful, I wanted people to like me, and I thought the smaller I was, the less horrid of me there would be. Participant008: The eating disorder services are very much focused on let's kind of get you to a healthy weight, rather than actually like what's causing the behaviours. I think actually a lot of people with eating disorders have kind of underlying, whether it's personality disorder or a different kind of mental health difficulty, I think there's not, just from my experience anyway, there's not much actual support with like the mental side of things, if that makes sense.

### 3.1 Theme 1: Navigating Diagnosis: The Quest for Understanding and Effective Support

This theme encapsulates the service‐user perspective that receiving a diagnosis is important in accessing the “right care” and facilitates a deeper understanding of the self. Some interviewees felt clinicians were "*not interested in diagnosing properly [001]*,” leading to a feeling that it “*devalued my situation [001]*.” Others suggested that when clinicians did not accurately identify their difficulties, they felt a sense of perpetual fighting for oneself, “*it's always been fighting, fighting, fighting for myself, for answers and the right help [003]*.”

Those who did receive a diagnosis often spoke of the lack of information provided following diagnosis. Many discussed doing research into their own difficulties was important in *‘being able to see how it played into my eating disorder, and what I was gaining from my eating disorder [008]”*, and *“understanding the links to my childhood trauma and why it might've started in the first place [008].”* Many felt that understanding more about the ED and EUPD comorbidity could help foster self‐compassion and identify why specific triggers feel more difficult for them. Most interviewees felt their difficulties were identified following significant delays, which had led to the majority seeking out other routes of care to gain ‘answers’ (such as private practice).

### 3.2 Theme 2: Experiences of Siloed Care: “It's Either the EUPD or the ED”: Experiences of Disconnect and Misplaced Focus

Most interviewees felt they needed to pick one presenting need to work on, rather than having a holistic overview of their difficulties. They reflected a separated stance on their difficulties echoed throughout the treatment journey, *“you either treat the eating disorder or you treat the personality disorder, rather than it being a holistic thing [008]*,” with experiences of care feeling disjointed.

Interviewees noted a conflict between the interlinked nature of their difficulties and the notion that *“it feels like it's one or the other, and you sort of have to prioritise which [006]*.” Given the isolated nature in which the EUPD and ED comorbidity has been viewed through care‐seeking experiences, a large proportion of interviewees shared a feeling of *“not knowing how to speak about it [004]”* with relation to the lens of comorbidity, particularly when the service focus was on solely the EUPD or the ED alone. Interviewees felt their ED comorbidity had been touched upon only briefly at assessments, leading to a perceived disconnect between service‐user and clinician focus.

Interviewees perceived an overemphasis on managing symptoms rather than understanding the cause or interplay between their EUPD and ED. They spoke about the importance of understanding the “*why*” of their difficulties in aiding them to implement the coping skills beyond the point of crisis. Many acknowledged timing of this work was paramount to ensure stability and safety. Nevertheless, most service‐users noted the often‐neglected in‐depth exploration of shared underlying factors between EUPD and ED, such as childhood trauma. A similar pattern was observed within experiences of ED‐specific physical monitoring, with interviewees perceiving an overemphasis on the physical manifestations of their ED, neglecting psychological mechanisms.

Interviewees felt that professionals views often conflicted with their own, and that their eating difficulties were often *“wrapped up all in one as the personality disorder [001]*.” Interviewees reflected that *“it feels separate [the ED] to what I'm doing in my service, but for me, it's not [004]*.” Most interviewees expressed it would have been helpful to have their comorbidity explored in greater depth and in a more holistic manner.

### 3.3 Theme 3: Crisis as a Catalyst for Support: “You Have to Be Bad Enough to Get Care”

A pervasive narrative was that hitting crisis point often resulted in receiving care. Over half of the interviewees received a diagnosis or care for their difficulties once they had presented in hospital. Similarly, those who had not yet received intervention for their ED felt that *“…with the care, you have to be bad enough to get care, and I think I've often not been bad enough. it kind of then, well, badly, motivates you to want to get worse so you'll get help [009]*.” One interviewee said *“The ED team said no further input, as I was under complex needs, so the only sort of monitoring I get is if I end up at hospital. That means I have to end up in hospital for that to happen [006]*.” Interviewees noted that reaching crisis point can disrupt treatment continuity in the community and prolong recovery.

Interviewees expressed anger around their difficulties being considered at the late stage of crisis. One reflected that *“to have to get worse before the input really comes along, can be quite traumatising [001]”*. Interviewees described reaching crisis point for either EUPD or ED‐specific difficulties, which led to an increased provision of care and *‘being taken seriously [001]*.”

### 3.4 Theme 4: “Lost in the System”

Several interviewees highlighted the lengthy waiting times endured and feeling “*bounced around [003]*” services. Interviewees spoke about the need to “*be knowledgeable about services, and stand your ground, or you disappear [002]*,” “*you fall off the radar [009]*.” Difficulty in navigating the system and *“knowing how it works [009]”* were voiced by several interviewees.

Interviewees pondered whether navigating the system was exacerbated by clinician uncertainty around dealing with “complexity”: “*You get lost in the system because you're a complicated case…people don't know what to do. they'll say you're under their care, but you don't feel as though you are. So it kind of ticks a box [003],” “they say you're a chronic patient or a revolving door patient. and I think then professionals treat you very differently [007]*.” Uncertainty was also observed at the service‐level, which led interviewees to feel confused about the responsibilities of different services. One interviewee expressed feeling *“stuck in red tape [002]*.”

While most interviewees appreciated the obstacle of waiting times, they felt being bounced around “*exacerbated problems [003]*.” Interviewees suggested increased communication between services, alongside information about why a service was most appropriate for them, would have been beneficial.

### 3.5 Theme 5: Examples of Meaningful Care

Interviewees generally felt clinicians they had encountered were caring and empathetic. Interviewees who did have keyworkers, or consistent contact with a clinician, highlighted the positive impact of such consistency in helping them to navigate the system and build trust. Service‐specific examples of good care included the recognised knowledge and expertise surrounding physical monitoring in EDs.

Those within the Complex Needs Service highlighted the collaborative and person‐centred nature of the assessment, “*They explained how it works, the groups… it was good to be able to speak through it and like sort of decide together as well [006]*.” Moreover, while interviewees did not feel eating difficulties were explored in‐depth, several commented upon the detailed and “*holistic [007]*” nature of the assessment. Poignant aspects of intervention included the learning about the causes of EUPD, “*learning about the biopsychosocial model [009]*,” and access to therapeutic groups. Two interviewees expressed hope that they could bring eating difficulties to these groups in the future. Several interviewees found peer support workers helpful for instilling hope in recovery.

### 3.6 Theme 6: Need for an Integrated Approach: “It All Exists Within Me”

Some described their eating difficulties as a “*coping*” or “*control strategy* [007, 005]” for intense emotions, while others described EUPD and ED as “*very much coexisting hand in hand, and exacerbating each other* [001].” One interviewee stated “*I wanted people to like me. I thought the smaller I was, the less horrid of me there would be* [002],” with several others acknowledging the role of childhood trauma in the comorbidity between EUPD and ED. A more holistic approach to comorbid difficulties was perceived to “*speed up the recovery process [007]*.”

Most interviewees valued learning more about their difficulties and the interplay between the two, however acknowledge much of this information was gained through independent research. They discussed the importance of “*talking more about comorbidity [005]*,” so “*people in the future can feel like everything they struggle with matters, and no one thing matters more than the other [004]*.” Some said having clinicians “touch in” on aspects of their comorbidity, even if not the main treatment priority, would be helpful. Interviewees highlighted the fragmented nature of services, which separate EUPD and ED from one another, which conflicted with their perspective that “*it all exists within me* [006].” Some interviewees further highlighted the importance of a thorough assessment of needs, “*I guess for me…exploring the difficulties fully in the beginning to establish whether it is more part of the emotional regulation, or whether it's a separate diagnosis. I think more could be done there because I shouldn't have had to of fought for myself like I did* [001].” The utility of care planning for individualised presenting needs was emphasised by one interviewee, to move beyond prioritising physical monitoring alone for ED.

## 4 Discussion

This project aimed to explore how mental health service experiences can be improved at a local level for individuals with comorbid EUPD and an ED. Specifically, three objectives were addressed with main findings summarised alongside key implications:

1. To understand the service‐user experience of seeking, accessing and receiving care
  Service‐users reported long waiting times and experiences of fragmented care, with many often feeling compelled to prioritise diagnostic‐specific support for either their EUPD or ED. This was perceived to conflict with their personal goals. Delayed recognition of difficulties in the sample, with support often only accessed at crisis points, highlighted gaps between local practice and national guidance, which recommend joint‐working for individuals presenting with comorbid needs (National Colloborating Centre for Mental Health 2019). These findings underscore the need for adapted service models that incorporate patient perspectives and provide timely, coordinated support.
2. To identify any areas of unmet need for individuals with EUPD and an ED
  Comorbidity was frequently overlooked in the assessment and treatment of these service‐users. Within the sample, some viewed EUPD‐related difficulties as the main area of intervention, while others perceived a bidirectional relationship between their comorbidity, with some viewing EUPD and ED as “exacerbating one another”. These experiences align with prior research demonstrating poorer outcomes for comorbid presentations when traditional evidence‐based models are not adapted for addressing comorbidity (Denning et al. 2023; Federici 2010; Park et al. 2014; Rowe et al. 2008; Thompson‐Brenner et al. 2016). Combining elements of evidence‐based models for both EUPD and an ED (e.g. DBT with incorporation of CBT modules for ED's around nutrition/body image, or CBT for ED's with incorporated distress tolerance modules) have demonstrated preferential outcomes (Ben‐Porath et al. 2009; Chen et al. 2008; Federici and Wisniewski 2013; Kröger et al. 2010; Palmer et al. 2003; Salbach‐Andrae et al. 2008).
  Taken together, the wider literature and findings of this study highlight the need for interventions that cater towards comorbidity. Specifically, participants valued psychoeducation about the interplay between EUPD and ED, indicating the need for integration within the assessment and treatment of this population.
3. To create recommendations for improving care for individuals with EUPD and an ED.

Our findings highlight an imperative need for inclusion of patient preferences and valued as part of effective clinical decision‐making, alongside robust assessment of needs. The overall results support person‐centred, integrative approaches consistent with the NHS Long‐Term Plan (2019) Participants within the sample also experienced extended waiting times, which conflict with the NHS Confederation's notion that there is ‘No wrong Door’ for individuals accessing support, rather, an open model enables service‐users to access the care needed, irrespective of where their first point of contact was (Bell and Pollard, 2022).

Most participants were happy with individual service provision, however felt it would be beneficial for clinicians to acknowledge and explore comorbidity from the outset of assessment and treatment to tailor care to individual needs. This view was particularly shared by those who had received previous physical or psychiatric admissions for their ED and who perceived disordered eating as a coping strategy for emotional dysregulation, which is seen as the underlying unmet need. For some, continued support within existing services may suffice; others may benefit from integrated evidence‐based interventions, such as adapted models seen in the wider literature, such as adapted DBT or CBT. This may be supported by multi‐service consultation at a local level.

Specific recommendations for improving care for this population, within Buckinghamshire community mental health teams can be found in Table 3, which could be relevant for other areas in the UK. Figure 2 details a proposed framework for improving care at a local level for individuals with comorbid EUPD/ED across community mental health services.

**Table 3:** Full list of recommendations for improving care across pathways for individuals with comorbid EUPD and ED across Buckinghamshire Community Mental Health Services.

Area	Specific recommendations
Assessment of needs	1. Service‐users should not be referred to pathways based solely on diagnosis. A thorough assessment of needs should be completed.
	2. Referral to the CEN service may be indicated when eating behaviours appear to be driven primarily by emotional dysregulation and present alongside other coping behaviours alone and used as a coping strategy.
	3. In cases where the ED and EUPD appear to co‐occur and/or persist, consultation between the A‐ED and CEN services should seek to care plan for both needs accordingly.
	4. Provide a person‐centred assessment where service‐user preferences and treatment goals are established and used to design an individualised, formulation‐led care plan.
	5. If referral to a differential team is warranted, provide the rationale and information about the Community Mental Health Team pathways, to aid service‐user understanding.
	6. A clear agreement around how physical wellbeing will be monitored pre‐emptively should be discussed collaboratively with the service‐user and a care plan created. ▪ There should be a clear rationale for the frequency of monitoring, with good communication between professionals and the service‐user.
	7. A low BMI and ability to work psychologically should be considered on an individual basis. A low BMI should not exclude a service‐user from accessing evidence‐based care.
Treatment	8. Following assessment, a stepped level of psychological intervention can be referred to, which suggests that universally: ▪ All patients with EUPD/ED comorbidity should receive ongoing risk assessment ▪ There are clear timeframes to regularly review ED and EUPD symptoms, using a functional analysis. ▪ All patients with EUPD/ED comorbidity should receive a psychoeducation package, which enables understanding around the shared aetiology, maintenance and risk factors between both disorders.
	9. For service‐users with a trauma history, and/or the eating disorder is not primarily driven by emotional dysregulation alone, consider targeted support where: ▪ Treatment within the service actively acknowledges the comorbidity and applies it to the evidence‐based therapeutic framework with the service‐user. ▪ There is ongoing consultation between the A‐ED and CEN pathways to facilitate incorporation of comorbidity within evidence‐based treatment. ▪ Trauma needs are considered and incorporated in treatment‐planning and decision‐making. For individuals with trauma histories, there may be a need to spend further time exploring the historic contributing factors of difficulties, to aid implementation of coping skills.
	10. For individuals with persisting EUPD and ED symptoms, or with therapy‐interfering behaviours, access to services should not be denied based on BMI, and/or suicidal risk alone. ▪ The possibility of a service‐user accessing multiple services should be explored, with a thorough care package finalised between services and the service‐user.
	11. For individuals with suspected ED and EUPD, consider access to peer support groups, or peer support workers and/or liaison with a consistent clinician, such as a care coordinator.
Whole of care	12. To consider specialist staff from the A‐ED or CEN pathway offering consultation to other community teams, particularly during transition to another team.
	13. To understand the level of staff and professional understanding surrounding the EUPD/ED comorbidity and facilitate ongoing training and supervision to accommodate continuing practice and development.
	14. Services to recognise that individuals presenting with both EUPD and ED are more likely to have had or have a negative experience of care, or experiences services as disjointed. Ensure that there are regular reviews of care, taking into account patient feedback.
	15. For community mental health teams and/or relevant services treating individuals with EUPD and ED, to be able to bring clinical discussions to other teams consultation and forum slots where necessary, in order to empower staff to gain specialist knowledge to integrate into clinical care.

Abbreviations: A‐ED, Adults Eating Disorders; BMI, Body Mass Index; CEN, Complex Emotional Needs; ED, Eating Disorder; EUPD, Emotionally Unstable Personality Disorder.

**Figure 2:**
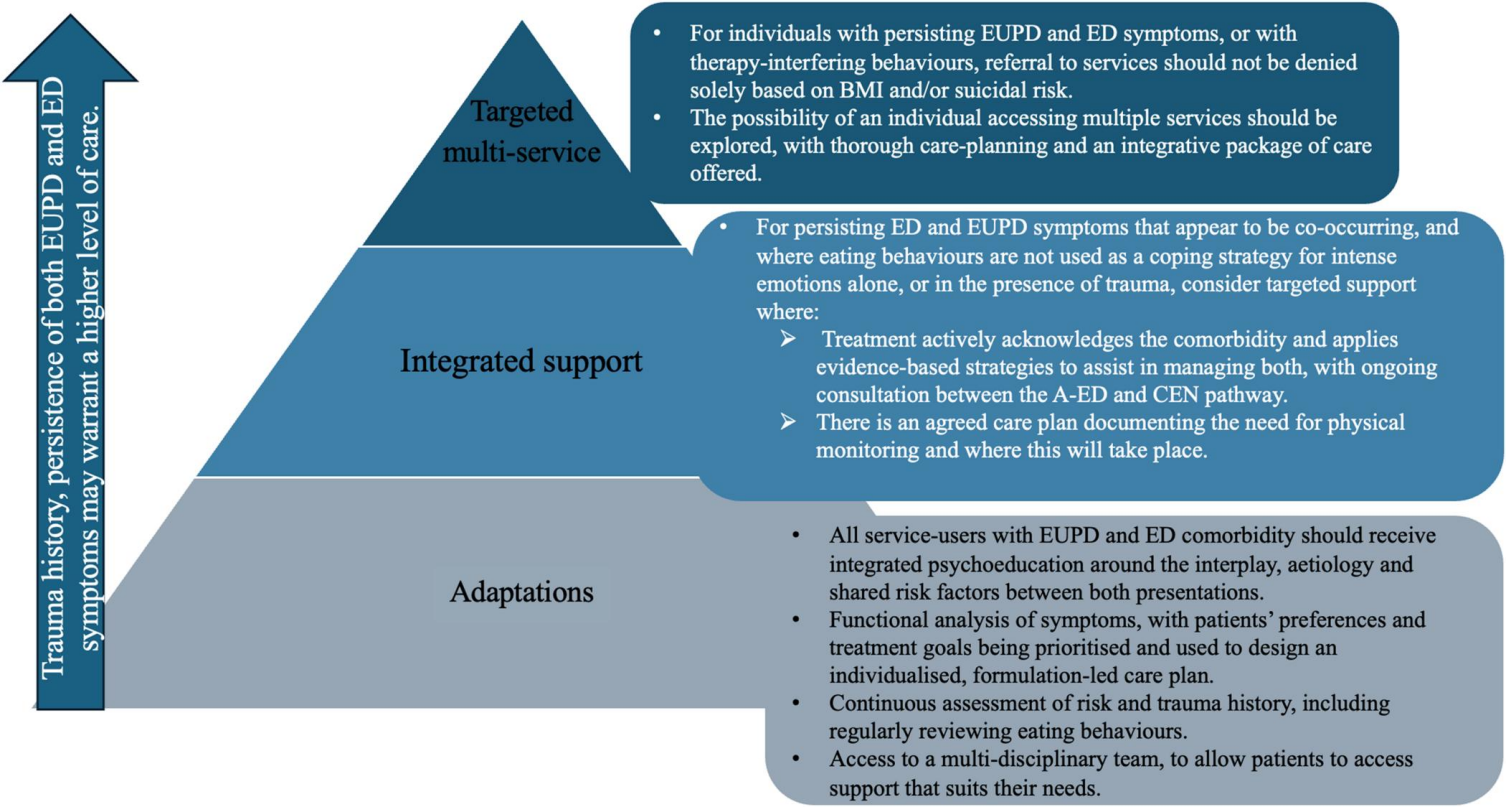
Proposed Framework for Improving Care for Service‐Users with Comorbid EUPD and an ED. A‐ED, Adult Eating Disorders; BMI, Body Mass Index; CEN, Complex Emotional Needs; ED, Eating Disorder; EUPD, Emotionally Unstable Personality Disorder.

This project provides some evidence that the Biosocial Model (Linehan 1993) Wisniewski and Kelly (2003) can be applied to comorbid EUPD and ED. However, the data also suggested some interviewees viewed their ED as a bidirectional influence alongside their EUPD, suggesting this model may be somewhat reductionistic. While the Biosocial Model remains a helpful framework for considering the EUPD/ED interplay, this project emphasises the importance of matching intervention to a thorough clinical assessment of need and formulation.

### 4.1 Strengths, Limitations and Next Steps

The emphasis upon experts by experience was a strength of the present project, particularly given very few studies have previously examined patient perceptions of treatment or experiences of care. The rich dataset reveals important insights into the interplay between EUPD and EDs from a lived experience perspective, which in turn could help clinicians understand what therapeutic mechanisms are most meaningful for service users.

Our sample size was small (although similar to others using similar qualitative methodologies), with all patients identifying as White British females. This hinders extrapolation of results to other populations and service contexts, although our experience of engagement with service leads nationally suggest similar service delivery challenges for addressing the needs of EUPD/ED comorbidity. Moreover, the methodological approach, TA, is highly dependent on interpretation, which can lead to variation in theme identification and analysis, further impacting the replicability of findings.

It is paramount to analyse the efficacy and feasibility of proposed recommendations to ascertain improvements in care. To understand the impact upon resourcing, it would be vital to assess the proportion of service users receiving differing levels of care and to monitor associated changes in outcomes across A‐ED and CEN pathways. The relevance of these recommendations for EUPD/ED populations outside of OHFT could also be explored.

## 5 Conclusions

This study highlights substantial gaps in the provision of care for individuals with comorbid EUPD and ED. Participants often described protracted waiting times, fragmented pathways and pressures to prioritise one diagnosis over the other, typically resulting in delayed or crisis‐driven access to support. Comorbidity was frequently insufficiently recognised within assessment and treatment processes. These findings align with a growing evidence base indicating that traditional, single‐diagnosis approaches yield poorer outcomes for this group, whereas integrated and adapted interventions demonstrate greater effectiveness.

Collectively, the results underscore the need for timely, coordinated and person‐centred models of care that incorporate patient perspective and acknowledge the complexity of comorbid presentations. Participants valued psychoeducation and collaborative clinical decision‐making. Recommendations derived from the results of this study provide locally relevant, evidence‐informed strategies for improving care pathways. Overall, this study calls for a shift towards integrated, formulation‐driven approaches that can more effectively meet the needs of this population within community mental health services.

## Consent

Informed consent was gained prior to each interview, and gathered electronically. Consent was given verbally at the beginning of each interview.

## Conflicts of Interest

Four of the authors (MD, JH, JS, LS) were clinicians who worked across the clinical services, whilst this project was conducted. Attempts were made to be objective in the interpretation of all study findings and within this article. Nevertheless, we acknowledge that their involvement may add an element of bias. The other authors declare no conflicts of interest.

## Data Availability

The data associated with this research is not available, as service‐users did not provide consent for data sharing, and there is a possibility for confidentiality to be violated by sharing the interview transcripts. The interview schedule is included in the appendix.
